# Post-ART Symptoms Were Not the Problem: A Qualitative Study on Adherence to ART in HIV-Infected Patients in a Mozambican Rural Hospital

**DOI:** 10.1371/journal.pone.0137336

**Published:** 2015-09-02

**Authors:** Maria Maixenchs, Helena Boene, Rui Anselmo, Carolina Mindu, Pedro Alonso, Clara Menéndez, Eusébio Macete, Robert Pool, Emílio Letang, Denise Naniche, Khátia Munguambe

**Affiliations:** 1 Centro de Investigação em Saúde de Manhiça (CISM), Manhiça, Mozambique; 2 ISGlobal, Barcelona Ctr. Int. Health Res. (CRESIB), Hospital Clínic—Universitat de Barcelona, Barcelona, Spain; 3 Faculty of Medicine, Eduardo Mondlane University, Maputo, Mozambique; 4 Centre for Social Science and Global Health, University of Amsterdam, Amsterdam, The Netherlands; 5 Swiss Tropical and Public Health Institute, Basel, Switzerland; University of Athens, Medical School, GREECE

## Abstract

**Objective:**

The objective of this qualitative study was to explore how clinical symptoms may affect adherence to antiretroviral therapy (ART) in HIV patients, and to explore factors, perceptions and attitudes related to adherence to therapy.

**Design:**

A qualitative study was carried out in the context of the prospective cohort study **“**Evaluation of Immune Reconstitution Following Initiation of Highly Active Antiretroviral Treatment in Manhiça, Mozambique”. In-depth Interviews were conducted twice in a sub-sample of the study cohort (51 participants), at six-month intervals.

**Results:**

Most participants (73%) knew that AIDS is a chronic disease and that ART does not cure it. Nine participants (18%) were non-adherent at some point and two (4%) abandoned ART. All participants but five reported having symptoms after starting ART, mainly attributed to pills needing time to act and body’s reaction to the treatment. In spite of the perceived severity of the symptoms, only two people reported they discontinued the treatment due to symptoms. Almost all participants reported feeling comfortable with the HIV clinic organization and procedures, but afraid of staff being hostile if they did not follow the *rules* or if the health worker visited their home. Family was one of the most important source of support according participants. Almost all participants with children said that a decisive factor to follow the treatment was the desire to be able to look after them.

**Conclusions:**

Experiencing symptoms after starting treatment was not a barrier to adherence to ART. Factors related to adherence included control measures set up by the health facility (exhaustive follow up, support, information) and family and community support. Indirect ART-related expenses did jeopardise adherence.

## Introduction

In 2007, an estimated 33 million people were infected with HIV, of which two-thirds were living in sub-Saharan Africa [1]. The number of new HIV infections has globally declined over the past decade but an estimated 35 million people world-wide were living with HIV by the end of 2013 [2].

In Mozambique, the national estimated HIV prevalence in adults was 12.5% in 2007 [1] and 10.8% in 2013 [2]. In the country, antiretroviral treatment (ART) coverage has increased from 88,211 (24%) in 2007 [3,4] to 456,055 (33%) in 2013 [2]. Adherence to ART in sub-Saharan Africa (SSA) is relatively good, with reported levels similar or even higher than those in the industrialised countries [5–8]. A meta-analysis comparing adherence in sub-Saharan Africa with North America estimated an overall adherence of 77% in SSA compared to 55% in the USA [9].

Barriers to adherence to ART have been identified at many levels, namely symptoms, health care settings and social context. Some studies in Africa have found symptoms during therapy to be a major barrier to ART adherence [9–12] while others have shown the contrary [13–15]. IRIS is a clinical worsening after initiation of ART, which occurs in up to 30% of patients [16] and could be a cause of many symptoms experienced after ART initiation. While such symptoms are physiopathologically related to an excessive response to pre-existing pathogens by the reconstituting immune system, patients could interpret them as a treatment failure. In South Africa, adults who experienced IRIS after initiating ART were slightly less adherent than those who did not experience IRIS. Despite the non-significant difference, the authors suggested that the resulting morbidity or the loss of confidence in ART may have reduced adherence [17].

Drivers of adherence include the desire to improve health [7], to live a similar life as those who are non-HIV infected [14,18,19] willingness to lead a productive life like other HIV-infected people [18] and fear of further physical deterioration [19]. These factors often outweigh the potential drawback of side effects and symptoms. Similarly, perceived health improvement after starting ART promotes adherence and trust in the medication [11,12,14,18–20].

Building rapport between patients and health providers has also been shown to influence adherence to ART [19,21–23]. Specifically, while the quality of counselling and education at the health facilities influences ART adherence positively [8,9,11,24], overloaded [24] and fatigued health providers [11], lack of empathy [11], or their stigmatization of patients [20] have negative effects on adherence. Additionally, inadequate healthcare settings such as overcrowded clinics [18], long waiting lines [8], delays [25], limited time for consultations [11,24] and medication stock out [14,25] have been shown to be significant barriers for adherence.

Social networks have a great influence supporting or discouraging adherence to treatment [20,26]. Apart from a single study in South Africa suggesting no associations between social support and adherence [27], there is an important body of evidence in favour of this association [7,12,14,18,19,26]. Ability to take care of one’s own children has also often been given as an additional important social motivator [11,14,18,19,21].

In spite of ART being free of charge, or subsidised, in most African countries, economic constraints are one of the main obstacles to adherence [9,24] with lack of food and transport being the most common barriers [7,8,11,12,18,20,21,26].

The objective of the present study was to explore how clinical symptoms may affect adherence to antiretroviral therapy (ART) in HIV patients, and to explore factors, perceptions and attitudes related to adherence to therapy.

## Methods

### Study setting

The study was conducted by the Centro de Investigação em Saúde de Manhiça (CISM) which collaborates with the HIV/AIDS National Control Programme with support and clinical studies since 2003.

The study took place in Manhiça District, Southern Mozambique, which had a population of 160,000 in 2007, 54% of which were under 20 years of age [28]. Population characteristics have been described in detail elsewhere [29,30].

HIV prevalence in women attending antenatal clinics at sentinel sites in Manhiça District was estimated to be 29% in 2007 [31], and the overall community-level HIV prevalence among adults was 39.9% in 2010 [32].

In the district, health services are provided by the Manhiça District Hospital (MDH), a 110-bed referral health facility. At the time of the study, MDH included the HIV/AIDS voluntary counselling and testing (VCT) service and an HIV clinic, through which ART was introduced in 2005. By 2008, 1,570 people were under ART according to the MDH registries.

Patients testing positive for HIV were referred for clinical visits and examinations, which were scheduled according to the Mozambican guidelines and included pre-treatment and regular clinical assessments at week 2, month 4 and every 6 months thereafter. These procedures have been described in detail elsewhere [33].

Patients fulfilling the Mozambican national established criteria to start ART [33] were referred for three additional pre-ART counselling visits about ART, opportunistic infections prophylaxis, adverse events, HIV prevention/transmission, nutrition and the importance of adherence. Stigma was also discussed. Each patient had to bring an adherence supporter who would endorse adherence and collect medication whenever the patient would be unable to do so. Talks on HIV/AIDS were regularly performed at the clinic by the health workers and a local voluntary group. A fieldworker was in charge of tracking non-adherent and defaulting patients in the community.

### Study Population

A prospective cohort study, “Evaluation of immune reconstitution following initiation of highly active antiretroviral treatment in Manhiça, Mozambique” (RITA study), was conducted between April 2006 and November 2008 by CISM to explore the incidence, clinical spectrum, and risk factors of IRIS among ART-naïve HIV-infected patients starting ART. In this study, 26.5% of patients starting ART developed IRIS with a median time of 62 days from ART initiation [16,33]. The present study involved a sub-sample of the RITA study cohort. Thirty-three percent of the participants had a clinical and immunological diagnosis compatible with IRIS within the first 4 months of ART (IRIS group) and the remaining did not (non-IRIS group).

### Definitions

#### Adherence to ART

The World Health Organization (WHO) defines adherence as the extent to which a person’s behaviour–e.g. taking medication- corresponds with agreed recommendations by a health care provider [34]. However, in this study, the definition provided by patients constitutes the source for defining adherence. For them, adherence meant not missing a single dose regardless of their attendance to the scheduled clinical visits. The recalled period considered was from ART initiation until the date of the interview.

#### Immune reconstitution inflammatory syndrome (IRIS)

To define IRIS, we used the criteria proposed by French *et al*. [35]: An abrupt clinical worsening of an existing condition (“paradoxical” IRIS) or new presentation of a previously unknown OI (“unmasking” IRIS) following ART initiation, and either with a concomitant reduction of at least 1 log_10_ of HIV-1 RNA levels at the time of IRIS or with 2 of the 3 minor criteria: (1) increased CD4 count after ART, (2) increase in an immune response specific to the relevant pathogen, (3) spontaneous resolution of disease [16,35]

### Methods

This qualitative study took place between July 2007 and June 2008. Sixty prospectively and purposively selected patients were invited to participate in in-depth interviews. The following were the inclusion criteria: ≥ 18 years old, > 8 months on ART, participant of the RITA study and willing to give informed consent.

Data were collected by two trained Mozambican social scientists. Each participant was invited to be interviewed twice, with a six-month interval. The interviews followed a topic guide exploring (i) participants´ knowledge about HIV/AIDS and ART, (ii) their experiences regarding symptoms, (iii) their perceptions and attitudes regarding adherence, and (iv) the role of the health facility, social dynamics, stigma and economic factors on dealing with symptoms and adherence.

Interviews were conducted in Changana or Portuguese, according to participant preference, and took about one hour each. Participants chose the interview location, either at home or at the clinic according to their preferences. Interviews were transcribed in Portuguese. In five cases, no permission was given for recording and detailed notes were taken.

Transcribed material was coded using NVivo 2 and later NVivo 10 (QSR International Pty Ltd.). Coding entailed identifying passages of text related to the topics of interest and labelling them with specific themes and sub-themes. First, the broad themes were based on the general research question: “Do symptoms and/or economics, social dynamics, health care setting influence adherence?” As transcripts were gradually imported into the project and thoroughly read, codes were refined, added or eliminated, depending on their relevance to the emergent data. By grouping together similarly coded text, discussions were built around each theme and around links between themes and sub themes, and from these discussions, the main conclusions emerged.

### Ethical considerations

The study was approved by the National Committee on Health Bioethics of Mozambique (Ref.34/CNBS/07) and the Hospital Clinic of Barcelona Ethics Committee (Spain). Written informed consent was obtained from all participants prior to interviews. Additionally, verbal, recorded consent was obtained for recording the interviews.

All data were managed based on unique identification numbers so as to guarantee patients’ confidentiality.

## Results

### Study participants

Out of 60 patients contacted, 51 were interviewed in the first round of data collection and 46 of these were interviewed again after a minimum of six months, totalling 97 interviews. Reasons for non-participation are summarized in Table 1.Main characteristics of study participants are summarized in Table 2.

**Table 1:** Reasons for non-participation.

Interview round	Reasons for non-participation	n	Subtotal (%)
**First round**	Selected participants	60	
	Declined	4	7%
	Working in South Africa	2	3%
	Internal migration	2	3%
	Died	1	2%
**Second round**	Participants	51	
	Working in South Africa	1	2%
	Travelling	1	2%
	Not found	1	2%
	Died	2	4%

**Table 2:** Participant characteristics.

		*n (51)*	*%*
**Sex**	Male	18	35%
	Female	33	65%
**Age group, years**	20–29	13	25%
	30–39	18	36%
	40–49	14	27%
	50–59	4	8%
	60–69	2	4%
**Marital status**	Single	14	27%
	Married/Living with partner	24	47%
	Divorced/Separated	4	8%
	Widower/Widow	9	18%
**Child bearing status**	With Children	42	82%
	Childless	9	18%
**Income**	Regular income	18	35%
	Irregular earnings	16	31%
	No income	17	34%
**HIV WHO Stage**	I-II	11	22%
	III-IV	40	78%
**CD4 cell count (cells/ μL) at ART initiation**	<100	28	55%
	100–200	12	23%
	>200	11	22%
**Baseline Viral load** Median (IQR)	5.06 (2.3–5.81)	NA	NA
**IRIS**	IRIS case	17	33%
	Non-IRIS case	34	67%
**ART Regimen**	d4T/3TC/NVP	28	55%
	AZT/3TC/NVP	4	8%
	d4T/3TC/EFV	8	15%
	AZT/3TC/EFV	11	22%
**Self-perceived ART**	Adherent	40	78%
**Adherence** ^**1**^	Non adherent at some point	9	18%
	Abandon	2	4%

^1^According to participants. Adherence meaning not missing a single dose.

### Views on ART

Most participants (73%) knew that AIDS is a chronic disease and that ART does not cure it. They were also aware that if they did not adhere to treatment the disease would reappear with more strength, new symptoms could emerge and that they could even die.

*“It’s been said that (the virus) doesn’t die*. *It falls asleep*. *If you stop taking the pills the disease starts again*. *You get sick*, *without strength*. *You can even die…*. *The pills make the virus drunk*. *When you stop taking the pills*, *the virus wakes up*.*”* (Female participant, 34 years old)

Awareness of the importance of adherence to treatment was evidenced by the sense of guilt expressed by the 9 self-reported non-adherent participants (4/17 from the IRIS-group and 5/34 from the non-IRIS group) and the 2 who reported to have abandoned the treatment, as they referred to their non-adherent behaviour as “failure” and they emphasised that it would never happen again.

*“I started treatment in November 2006 and I just failed now*. *(…) I have to come back to the clinic to pick up the pills*, *I will come back*. *Next Monday I will go to the clinic*. *I didn’t stop definitely*, *I will come back*.” (Male, 41 years old)

All participants considered ART effective, despite of it not curing HIV. The terms “cure” and “getting better” were used ambiguously in most of the interviews, and often linked to improvement rather than actual cure. Patients reported feeling healed, comparing their current well-being to how they felt before starting ART. However, when probed about the length of the treatment, most of them did not know whether and when the treatment was going to finish. They assumed that the doctor would inform them and that it depended on each case.

Main advantages reported by participants included ART permitting survival, improving health and allowing a normal daily life (working and taking care of children and family), and ART being free of charge.

*“Since the day I started coming to the hospital I am better*, *I work*, *I do everything*. *I have come today because I feel backache because I carried very heavy things*. *I am feeling so well that I do heavy work*.*”* (Female participant, 43 years old)

Regarding disadvantages of ART, participants referred to perceived side effects but mostly linked to issues relating to food. Almost all participants reported having to take the pills with food because “the pills are very heavy”. Surprisingly, the fact that treatment has to be taken every day was seldom mentioned as a disadvantage.

### The role of perceived symptoms on adherence

All participants reported having symptoms prior to ART initiation, as most of them were in advanced stages at first presentation to the Clinic. Most symptoms were gradually cleared after starting ART and patients reported feeling better within 2–3 months.

After starting ART, all but five of fifty-one participants reported new or persistent symptoms (90%). Of these, only four participants did not attribute them to the ART but to malaria, TB, AIDS and diet. Participants considered persisting symptoms just a matter of time before the pills would start to act. New symptoms were attributed to the body´s reaction to the treatment itself.

Since IRIS causes clinical worsening and accompanying symptoms, we analysed the perceived symptoms according to whether or not the participant had a diagnosis of IRIS (Table 3). Despite the diagnosis of IRIS, the kind or the perceived intensity of emergent symptoms barely differed between patients with an IRIS episode and patients without. The majority of participants perceived the symptoms experienced after starting ART as severe, not allowing them to perform their daily tasks. The majority explained they felt they were not improving, but it would be even worse if they stopped, according to the information received at the clinic. Witnessing other patients improve also encouraged adherence.

The main symptoms reported by participants are summarized in Table 3.

**Table 3:** Signs and symptoms reported by participants.

SIGN, SYMPTOM^1^	REPORTED BEFORE ART INITIATION		REPORTED AFTER ART INITIATION	
	IRIS GROUP	NON IRIS GROUP	IRIS GROUP	NON IRIS GROUP
Headache	Yes	Yes	Yes	Yes
Fever	Yes	Yes	Yes	Yes
Weakness	Yes	Yes	Yes	Yes
Dizziness	Yes	Yes	Yes	Yes
Low blood pressure	No	No	Yes	No
Weight loss	Yes	Yes	Yes	No
Lack of appetite	Yes	Yes	Yes	No
Vomiting, nausea	Yes	Yes	Yes	Yes
Stomach-ache	Yes	Yes	Yes	Yes
Diarrhoea	Yes	Yes	Yes	Yes
Chest pain	Yes	Yes	Yes	Yes
Breathing difficulties	Yes	Yes	Yes	Yes
Cough, flu-like symptoms	Yes	Yes	Yes	Yes
Skin problems (skin rash, wounds)	Yes	Yes	Yes	Yes
Back, body and joint pain	Yes	Yes	Yes	Yes
Swelling (body, feet)	Yes	Yes	Yes	Yes
Lack of menstruation	Yes	No	Yes	No
STD like symptoms (e.g. genital ulcers, aching, vaginal discharge)	Yes	Yes	Yes	Yes
*“Herpes Zoster”*	No	No	Yes	No
*“Kaposi´s sarcoma”*	No	No	Yes	No
Others	Yes	Yes	Yes	Yes

^1^Perceived signs and symptoms reported by participants; STD: Sexually transmitted diseases

The IRIS patients presented a diversity of diagnoses (Table 4):

**Table 4:** Clinical characteristics of IRIS cases.

Patient IDI	Time to IRIS (days)	Diagnosis	IRIS Type	Outcome
4	36	Kaposi´s sarcoma	P	R
5	21	Kaposi´s sarcoma	P	R
8	34	Pneumocystis jirovecii pneumonia	P	R
10	39	TB lymphadenitis	P	R
11	64	Pulmonary TB/ Kaposi´s sarcoma	P/U	R
13	24	Genital Herpes	U	R
14^a^	126	Tinea corporis	U	R
15	11	Genital Herpes	U	R
17	76	Tinea corporis	U	R
19	8	Herpes zoster	U	R
26	76	Pulmonary TB	U	R
27	161	Kaposi´s Sarcoma	U	R
29	21	Impetigo	U	D (non-IR)
31	39	Interstitial pneumonitis	U	R
34	91	TB lymphadenitis	U	R
35	35	Milliary TB/ Kaposi´s sarcoma	U	R
36	56	Pleuro-pulmonary TB	U	R

^a^Probable case. The rest are confirmed cases. P: Paradoxical IRIS; U: Unmasking IRIS; R: Recovered; D (non-IR): Non-related IRIS death. Data abstracted from full table in Letang et al where all virological and immunological data is available [16].

The overall complete adherence (according to participant definition of adherence) among all participants was 78%.There seems to be no difference of self-perceived non adherence between IRIS and non-IRIS cases as 20% (4/17) of IRIS patients and 23% (7/34) of non-IRIS patients were not adherent. Only 2 participants reported discontinuing the treatment specifically because of the symptoms. One of them was from the IRIS-group and the other from the non-IRIS group.

When experiencing symptoms, most of the participants reported seeking care at the clinic, where the treatment regimen would be adjusted and further counselling given. Very few participants reported using traditional medicine after starting ART (4/51), probably because the clinic staff discouraged mixing traditional medicine with ARV drugs. However, this recommendation can also disfavour ART adherence, as evidenced by one participant who discontinued ART temporarily while trying traditional treatments.

*“When I started with the treatment*, *I felt a lot of pain*. *I cannot lie*, *I went to a traditional healer*. *He gave me treatment*. *I took it but the vomits and the swelling continued (*..*)*. *I stopped the pills (ART) for 2 months (…) I got worse*. *Then I went back to the clinic and I restarted taking the pills*.*”* (Female participant, 34 years old)

An important source of support for patients dealing with symptoms was the family, providing care, advice and emotional support.

### The role of the health facility setting on adherence

Patients attended regular, exhaustive follow-up visits at the clinic, during which sufficient time was reserved for them. They were told not to use other health services, and to come back if they did not feel well. A field worker traced defaulters at home. Regardless of being a defaulter or not, all patients were visited by the field worker to confirm scheduled visits.

Participants explained that “following hospital rules” would help them gain weight and strength gradually and consequently, improve their health status.

Almost all participants reported feeling comfortable with the clinic´s procedures and organization. However, they hoped for improvement in the delays during clinical visits and collection of medication, which they attributed to a lack of personnel and to an increase in the number of patients. Participants reported they would prefer collecting treatment every 2 to 3 months instead of every month due to the long distances between their homes and the clinic. Very few participants complained about the time lag between diagnosis and initiation of ART.

Participants reported that when they “did not follow the hospital rules” or missed a visit they would rather wait for a while before going back because they feared that staff would be angry. Most participants reported feeling uncomfortable when receiving home visits from the clinic, fearing that the community would realize their HIV status or concerned about having done something wrong.

*“He (the field worker) asked the neighbours where my house was and they pointed at it*. *Just by seeing someone driving a motorbike*, *they (the neighbours) can tell that person comes from the clinic*, *they like to tease you*. *They laugh at me*, *when you walk on the street; they say*, *"She has AIDS*! *She has AIDS*!*” I don’t like when it happens*.*”* (Female Participant, 31 years old)

There were participants, however, who reported feeling grateful when the clinic field worker came to their house because it showed that the clinic cared about them.

Very few participants reported that advice given at the clinic had not been understood. Even the self-reported non-adherent individuals believed in the need of following the treatment schedule rigorously, and understood the consequences of not doing so.

### The role of family and social context on adherence

Emotional and financial support, given by partners, family, close neighbours and friends were a key factors for adherence, especially during the first weeks of treatment, when symptoms arose. Such support guaranteed their basic needs such as money for transport and food, housework and childcare, as well as useful advice and encouragement when participants become weak.

Almost all participants with children said that a decisive factor to adhere to the treatment was the desire to be able to look after their children.

*I have a family; I have to take care of my wife*, *of my children*. *They (the children) have to continue going to school*. *If I am not here*, *nobody will take care of them*.*”* (Male participant, 45 years old)

They even reported that they informed their children that they were ill, not necessarily revealing their HIV status. Those participants explained that this created an environment of trust within the family, and involved the children in simple tasks, such as notifying them when it is time to take the medicines. Participants reported that, as a result, children felt useful and valued, while leaving parents feeling cared of and loved.

When talking about partners, participants reported that the relationship itself was a great source of support if both were HIV positive. In serodiscordant couples, with the male partner HIV positive, the woman would adopt the role of carer. In contrast, there were reports from female participants of being abandoned by their partner when they revealed their HIV status, sometimes influenced by family, friends and neighbours.

*“As soon as I became very sick*, *my husband left me*. *I couldn’t even get out of bed*. *He left me because I was sick*. *So it’s my family that helps me*, *my relatives*. *He called me a few days ago*. *He’s in South Africa*, *and he is sick*. *I’ll go because he’s my husband*, *I had five children with him*. *So I can’t abandon him like he abandoned me*. *When he gets sick I worry*.*”* (Female participant, 41 years old)

Some participants felt isolated, prosecuted and humiliated when their HIV status was suspected or disclosed to the community. Nevertheless, participants said they were committed to continuing the treatment and going to the clinic without fear of being seen there. At the same time, they developed strategies to avoid the disclosure by hiding their medication or not taking it in front of other people. The fact that they were free to choose the time at which they took their pills, was a facilitator for this strategy.

Although ART was free, participants were aware of the importance of economic conditions, such as transport and food, in adherence. Three self-reported non-adherent individuals attributed lack of food as the reason for non-adherence. Participants working in subsistence farming did not consider missing one farming day as a problem. Among those with formal jobs, only one reported not having permission to leave his job as the reason for defaulting.

Unforeseen events at the family level also contributed to the occasional skipping of medication. For example, travelling to visit family and staying longer than expected, travelling without the pills or the impossibility of collecting the treatment on the scheduled day due to own or children´s illness.

## Discussion & Conclusion

This is one of the few studies exploring factors influencing adherence of patients with IRIS-related symptoms during the initial phases of ART rollout in sub-Saharan Africa. Although in 2 distinct clinical groups, almost all IRIS and non-IRIS, patients reported experiencing symptoms after initiating ART. Subsequent adherence did not seem to differ according to IRIS status. Experiencing symptoms did not lead participants to doubt therapeutic efficacy or to develop negative attitudes towards adherence to ART. Our qualitative assessment corroborates a quantitative study in South Africa suggesting minimal differences in ART adherence between IRIS and non-IRIS patients [17]

The clinic set-up played a major role in the adherence to ART. Information given, time dedicated to patients and solutions provided for patients experiencing symptoms made them feel comfortable. In contrast, the strong measures applied for tracking and tracing or the fear of staff being hostile when indications were not followed, made participants feel anxious and guilty for failing to “follow the hospital rules”. Thus, despite the apparent immediate positive effect on adherence, extremely close follow-up may have its down-side. Recent studies looking at factors related to adherence have stressed the need for special training regarding confidentiality and non-judgmental care [19,20] and the benefits of empathic attitudes.

Most importantly, stigma jeopardized adherence to treatment. According to participants, the risk of status disclosure to other family and community members was an important issue. However, willingness to stay healthy, particularly to be able to take care of children or maintain daily chores, prevailed. Efforts have to be made to reduce stigma, but as long as it exists, it needs to be dealt with adequately. In MDH, the HIV clinics became chronic diseases clinic in 2013. The extent to which this could have contributed to stigma reduction or improvements in adherence needs further evaluation. Innovative technologies, such as SMS mobile texting could be used to communicate with patients, circumventing risks of disclosing confidential information [36].

Barriers to attendance and adherence were often practical, such as costs involved in reaching the clinic, or illness of the patient or of a close relative. Recently, the community ART groups (CAG) have been suggested as a solution to these barriers. CAG have been implemented In Mozambique since 2012 as part of the National HIV strategy. In a study in Tete province, participants reported time and cost savings as the major benefit of the CAG model [37].

Our study had two main limitations. As a qualitative study, the sample size limits generalizability beyond the setting in this single clinic, which may also not be representative of the situation in other rural areas as it works closely with a major health research centre with strong presence in the area. However, as this clinic was the primary provider of ART in the district, this study can provide useful insights about potential barriers or facilitators for adherence to ART among adults in a rural area of Southern Mozambique.

Notably, this study was performed in an ART-naïve population in 2005–6 [16], which marked the beginning of the ART rollout in Manhiça District. Subsequently, ART coverage has increased in Mozambique and has hovered at approximately 50% since 2010 [38,39], leading to increased survival and higher community prevalence of HIV [40,41]. Because it is likely that a population’s perception of ART changes as ART coverage increases, this unique study provides a valuable tool for an insight of potential determinants of adherence that can be referred to in current and future studies.

In conclusion, experiencing symptoms after starting ART was not a barrier to adherence. This study found no important differences in adherence to ART between patients with IRIS and those without. Most participants were motivated by improved health and the care for children, and were encouraged by the clinic retention strategies. The main barrier to adherence was the cost involved in regularly attending the clinic and food expenses, perceived as essential for a proper recovery.

## References

[1] UNAIDS-WHO. Report on the global AIDS epidemic [Internet]. Global Summary. 2008. Available: http://www.unaids.org/sites/default/files/en/media/unaids/contentassets/dataimport/pub/globalreport/2008/jc1510_2008globalreport_en.pdf

[2] UNAIDS-WHO. The GAP report [Internet]. 2014. doi:ISBN 978-92-9253-062-4

[3] Republic of Mozambique. National AIDS Council. Universal Declaration of Commitment on HIV and AIDS Mozambique Progress Report for the United Nations General Assembly [Internet]. 2008. Available: http://data.unaids.org/pub/report/2008/mozambique_2008_country_progress_report_en.pdf

[4] UNAIDS/WHO. Epidemiological Fact Sheets on HIV and AIDS, Mozambique 2008 Update. [Internet]. 2008. Available: www.unaids.org/en/CountryResponses/Countries/mozambique.asp (accessed 14 June 2009)

[5] Orrell C, Bangsberg DR, Badri M, Wood R. Adherence is not a barrier to successful antiretroviral therapy in South Africa. AIDS. 2003;17: 1369–75. 10.1097/00002030-200306130-00011 12799558

[6] Ortego C, Huedo-Medina TB, Llorca J, Sevilla L, Santos P, Rodríguez E, et al Adherence to highly active antiretroviral therapy (HAART): a meta-analysis. AIDS Behav. 2011;15: 1381–96. 10.1007/s10461-011-9942-x 21468660

[7] Ware NC, Idoko J, Kaaya S, Biraro IA, Wyatt M a, Agbaji O, et al Explaining adherence success in sub-Saharan Africa: an ethnographic study. PLoS Med. 2009;6: e11 10.1371/journal.pmed.1000011 19175285 PMC2631046

[8] Hardon a P, Akurut D, Comoro C, Ekezie C, Irunde HF, Gerrits T, et al Hunger, waiting time and transport costs: time to confront challenges to ART adherence in Africa. AIDS Care. 2007;19: 658–65. 10.1080/09540120701244943 17505927

[9] Mills EJ, Nachega JB, Buchan I, Orbinski J, Attaran A, Singh S, et al Adherence to antiretroviral therapy in sub-Saharan Africa and North America: a meta-analysis. Jama. 2006;296: 679–90. 10.1001/jama.296.6.679 16896111

[10] Olowookere S, Fatiregun A. Prevalence and determinants of nonadherence to highly active antiretroviral therapy among people living with HIV/AIDS in Ibadan, Nigeri5a. J Infect Dev Ctries. 2008;2: 7–10. 10.3855/jidc.199 19745505

[11] Bezabhe WM, Chalmers L, Bereznicki LR, Peterson GM, Bimirew M a, Kassie DM. Barriers and facilitators of adherence to antiretroviral drug therapy and retention in care among adult HIV-positive patients: a qualitative study from Ethiopia. PLoS One. 2014;9: e97353 10.1371/journal.pone.0097353 24828585 PMC4020856

[12] Nyanzi-Wakholi B, Lara AM, Munderi P, Gilks C. The charms and challenges of antiretroviral therapy in Uganda: the DART experience. AIDS Care. 2012;24: 137–42. 10.1080/09540121.2011.596518 21777081

[13] Barfod T, Sørensen H, Nielsen H. “Simply forgot”is the most frequently stated reason for missed doses of HAART irrespective of degree of adherence. HIV Med. 2006; 285–290. 10.1111/j.1468-1293.2006.00387.x 16945072

[14] Lyimo R a, de Bruin M, van den Boogaard J, Hospers HJ, van der Ven A, Mushi D. Determinants of antiretroviral therapy adherence in northern Tanzania: a comprehensive picture from the patient perspective. BMC Public Health. 2012;12: 716 10.1186/1471-2458-12-716 22935331 PMC3488585

[15] Al-Dakkak I, Patel S, McCann E, Gadkari A, Prajapati G, Maiese EM. The impact of specific HIV treatment-related adverse events on adherence to antiretroviral therapy: a systematic review and meta-analysis. AIDS Care. 2013;25: 400–14. 10.1080/09540121.2012.712667 22908886 PMC3613968

[16] Letang E, Miró JM, Nhampossa T, Ayala E, Gascon J, Menéndez C, et al Incidence and predictors of immune reconstitution inflammatory syndrome in a rural area of Mozambique. PLoS One. 2011;6: e16946 10.1371/journal.pone.0016946 21386993 PMC3046140

[17] Nachega J, Morroni C. Impact of immune reconstitution inflammatory syndrome on antiretroviral therapy adherence. Patient Prefer Adherence. 2012;6: 887–891. 10.2147/ppa.s38897 23271897 PMC3526885

[18] Grant E, Logie D, Masura M, Gorman D, Murray S a. Factors facilitating and challenging access and adherence to antiretroviral therapy in a township in the Zambian Copperbelt: a qualitative study. AIDS Care. 2008;20: 1155–60. 10.1080/09540120701854634 19012078

[19] Watt MH, Maman S, Earp JA, Eng E, Setel PW, Golin CE, et al “It’s all the time in my mind”: facilitators of adherence to antiretroviral therapy in a Tanzanian setting. Soc Sci Med. Elsevier Ltd; 2009;68: 1793–800. 10.1016/j.socscimed.2009.02.037 19328609 PMC3530387

[20] Tomori C, Kennedy CE, Brahmbhatt H, Wagman J a, Mbwambo JK, Likindikoki S, et al Barriers and facilitators of retention in HIV care and treatment services in Iringa, Tanzania: the importance of socioeconomic and sociocultural factors. AIDS Care. 2014;26: 907–13. 10.1080/09540121.2013.861574 24279762

[21] Merten S, Kenter E, McKenzie O, Musheke M, Ntalasha H, Martin-Hilber A. Patient-reported barriers and drivers of adherence to antiretrovirals in sub-Saharan Africa: a meta-ethnography. Trop Med Int Health. 2010;15 Suppl 1: 16–33. 10.1111/j.1365-3156.2010.02510.x 20586957

[22] Vervoort S, Borleffs J. Adherence in antiretroviral therapy: a review of qualitative studies. Aids. 2007;21: 271–281. 10.1097/QAD.0b013e328011cb20 17255734

[23] Hardon A, Davey S, Gerrits T, Hodgkin C. From access to adherence: the challenges of antiretroviral treatment [Internet]. 2006. Available: http://wwwlive.who.int/entity/medicines/publications/challenges_arvtreatment15Aug2006.pdf

[24] Murray LK, Semrau K, McCurley E, Thea DM, Scott N, Mwiya M, et al Barriers to acceptance and adherence of antiretroviral therapy in urban Zambian women: a qualitative study. AIDS Care. 2009;21: 78–86. 10.1080/09540120802032643 19085223 PMC2821879

[25] Audu B, Morgan R, Rutter P. Qualitative exploration of the relationship between HIV/AIDS patients’ experiences of clinical services and treatment adherence at Maitama District Hospital, Abuja, Nigeria. AIDS Care. 2014;26: 270–3. 10.1080/09540121.2013.819410 23875980

[26] Roura M, Busza J, Wringe A. Barriers to sustaining antiretroviral treatment in Kisesa, Tanzania: a follow-up study to understand attrition from the antiretroviral program. AIDS Patient Care STDS. 2009;23 10.1089/apc.2008.0129 PMC2776987 19866538

[27] Ncama BP, McInerney P a, Bhengu BR, Corless IB, Wantland DJ, Nicholas PK, et al Social support and medication adherence in HIV disease in KwaZulu-Natal, South Africa. Int J Nurs Stud. 2008;45: 1757–63. 10.1016/j.ijnurstu.2008.06.006 18653188

[28] Instituto Nacional de Estadística. Tercer Recenseamento Geral da População e Habitação 2007. 2007.

[29] Sacoor C, Nhacolo A, Nhalungo D, Aponte JJ, Bassat Q, Augusto O, et al Profile: Manhiça Health Research Centre (Manhiça HDSS). Int J Epidemiol. 2013;42: 1309–18. 10.1093/ije/dyt148 24159076

[30] Nhacolo AQ, Nhalungo D a, Sacoor CN, Aponte JJ, Thompson R, Alonso P. Levels and trends of demographic indices in southern rural Mozambique: evidence from demographic surveillance in Manhiça district. BMC Public Health. 2006;6: 291 10.1186/1471-2458-6-291 17137494 PMC1712340

[31] República de Mozambique. Programa Nacional de Controle das ITS/HIV/SIDA. Ronda de Vigilância Epidemiológica do HIV de 2007(HIV 2007 Epidemiological Surveillance). 2008.

[32] González R, Munguambe K, Aponte J, Bavo C, Nhalungo D, Macete E, et al High HIV prevalence in a southern semi-rural area of Mozambique: a community-based survey. HIV Med. 2012;13: 581–8. 10.1111/j.1468-1293.2012.01018.x 22500780

[33] Letang E, Almeida J, Miró J. Predictors of Immune Reconstitution Inflammatory Syndrome–Associated With Kaposi Sarcoma in Mozambique: A Prospective Study. JAIDS. 2010;53: 589–597. 10.1097/QAI.0b013e3181bc476f 19801945

[34] WHO. Adherence to long-term therapies. Evidence for Action [Internet]. 2003. Available: http://www.who.int/chp/knowledge/publications/adherence_full_report.pdf

[35] French M a, Price P, Stone SF. Immune restoration disease after antiretroviral therapy. Aids. 2004;18: 1615–1627. 10.1097/01.aids.0000131375.21070.06 15280772

[36] Baranoski AS, Meuser E, Hardy H, Closson EF, Mimiaga MJ, Safren S a, et al Patient and provider perspectives on cellular phone-based technology to improve HIV treatment adherence. AIDS Care. 2014;26: 26–32. 10.1080/09540121.2013.802282 23742640 PMC4418185

[37] Rasschaert F, Decroo T, Remartinez D, Telfer B, Lessitala F, Biot M, et al Adapting a community-based ART delivery model to the patients’ needs: a mixed methods research in Tete, Mozambique. BMC Public Health. BMC Public Health; 2014;14: 364 10.1186/1471-2458-14-364 24735550 PMC3990026

[38] National AIDS Council—Republic of Mozambique. Global AIDS Response Progress Report for the Period 2010–2011 [Internet]. 2012. Available: http://www.unaids.org/sites/default/files/en/dataanalysis/knowyourresponse/countryprogressreports/2012countries/ce_MZ_Narrative_Report[1].pdf

[39] UNGASS. United Nations General Assambly Special Session on HIV and AIDS. Mozambique Progress Report 2008–2009. UNAIDS. 2010. [Internet]. 2010. Available: http://data.unaids.org/pub/Report/2010/mozambique_2010_country_progress_report_en.pdf

[40] González R, Augusto OJ, Munguambe K, Pierrat C, Pedro EN, Sacoor C, et al HIV Incidence and Spatial Clustering in a Rural Area of Southern Mozambique. PLoS One. 2015;10: e0132053 10.1371/journal.pone.0132053 26147473 PMC4493140

[41] Zaidi J, Grapsa E, Tanser F, Newell ML BT. Dramatic increases in HIV prevalence after scale-up of antiretroviral treatment: a longitudinal population-based HIV surveillance study in rural kwazulu-natal. AIDS. 2012;29: 997–1003.

